# Visualization of the Postoperative Position of the Hydrus^®^ Microstent Using Automatic 360° Gonioscopy

**DOI:** 10.3390/jcm13175333

**Published:** 2024-09-09

**Authors:** Julian Alexander Zimmermann, Sarah Kleemann, Jens Julian Storp, Cedric Weich, Ralph-Laurent Merté, Nicole Eter, Viktoria Constanze Brücher

**Affiliations:** Department of Ophthalmology, University of Muenster Medical Center, 48149 Muenster, Germany; sarah.kleemann@ukmuenster.de (S.K.); jensjulian.storp@ukmuenster.de (J.J.S.); cedric.weich@ukmuenster.de (C.W.); ralph-laurent.merte@ukmuenster.de (R.-L.M.); nicole.eter@ukmuenster.de (N.E.); viktoria.bruecher@ukmuenster.de (V.C.B.)

**Keywords:** minimally invasive glaucoma surgery, glaucoma, Hydrus Microstent, Gonioscopy, imaging

## Abstract

**Introduction**: Glaucoma, one leading cause of irreversible vision loss worldwide, is primarily caused by elevated intraocular pressure (IOP). Recently, minimally invasive glaucoma surgeries (MIGSs) have become popular due to their shorter surgical times, tissue-sparing nature, and faster recovery. One such MIGS, the Hydrus^®^ nickel–titanium alloy Microstent, helps lower IOP by improving aqueous humor outflow. The NIDEK GS-1 automated 360° gonioscope provides advanced imaging of the chamber angle for evaluation and documentation. The aim of this study was to test automated 360° gonioscopy for the detection of postoperative positional variations after Hydrus^®^ Microstent implantation. This study is the largest to date to evaluate post-op positioning of the Hydrus^®^ Microstent using the NIDEK GS-1. **Materials and Methods**: This study analyzed postoperative outcomes and stent location in eyes diagnosed with mild to moderate glaucoma that underwent Hydrus^®^ Microstent implantation with or without phacoemulsification. Patients with prior IOP-lowering surgery or vitrectomy were excluded. Analyses of the postoperative Hydrus^®^ Microstent position were based on the evaluation of automated 360° gonioscopy images. **Results**: Twenty-three eyes were included in the study, and all showed a reduction in IOP and a decrease in antiglaucomatous drop use postoperatively. Postoperative gonoscopic images showed variations in implant position. In all cases, the proximal inlet was clearly visible in the anterior chamber. The degree of protrusion into the anterior chamber was variable. The distal tip of the stent was visible behind the trabecular meshwork in Schlemm’s canal in five cases, in the anterior chamber in one case, and not visible in seven cases. In no case did postoperative alterations in the position of the implant lead to explantation. **Conclusions**: This study demonstrated that the Hydrus^®^ Microstent can effectively lower IOP even in the presence of postoperative positional variations. Automated 360° gonioscopy was found to be a useful tool to verify and document the postoperative position of the implant. Positional changes did not require device explantation in any of the cases evaluated.

## 1. Introduction

Glaucoma is one of the leading causes of irreversible vision loss worldwide. The disease can eventually cause blindness. The main risk factor for the progression of the disease is elevated intraocular pressure (IOP). Pathophysiologically, the progressive loss of retinal ganglion cells results in optic nerve damage and visual field loss. The most effective strategy to influence the progression of glaucoma is to lower IOP. The first line of treatment includes IOP-lowering eye drops and laser surgery [1,2,3]. If these approaches are not sufficient, several surgical procedures are available to correct the imbalance between aqueous humor production and outflow. The epithelium of the ciliary body is responsible for the production of aqueous humor, which drains mainly through the trabecular meshwork and Schlemm’s canal into the episcleral veins [1,2].

In recent years, there has been a trend in glaucoma surgery towards minimally invasive glaucoma surgery (MIGS) to lower intraocular pressure (IOP). These procedures are an alternative to traditional methods such as trabeculectomy, with the goal of achieving similar postoperative IOP reduction. These procedures are particularly suitable for individuals with mild or moderate primary open-angle glaucoma. The goals of MIGS are shorter surgical times, tissue-sparing procedures, ease of use, and shorter rehabilitation times. MIGS is often combined with cataract surgery, but is also available as a stand-alone treatment option [4,5,6,7].

The Hydrus^®^ Microstent (Ivantis Inc., Irvine, CA, USA) is one such ab-interno trabecular micro-bypass implant that is inserted through the trabecular meshwork into Schlemm’s canal. The microstent is made of nitinol, a metal alloy of nickel and titanium. The device is 8 mm long (major/minor axes: 292 µm/185 µm) [5].

The implant improves aqueous humor outflow through four laser-cut windows by bypassing the trabecular meshwork and dilating Schlemm’s canal by approximately 90°. The Hydrus^®^ Microstent is approved for use in conjunction with phacoemulsification [5].

Randomized controlled trials have demonstrated the efficacy of the Hydrus^®^ Microstent: clinical studies have shown a significant reduction in IOP after the implantation of the Hydrus^®^ Microstent compared to phacoemulsification alone [8].

Gonioscopy, the examination of the anterior chamber angle, plays an important role in the assessment of aqueous outflow facility. This examination helps to differentiate between open and closed angles and to assess their pigmentation as well as iris configuration [9]. Especially in glaucoma patients, gonioscopy is used to select patients for MIGS implantation based on anatomical conditions. Conventional gonioscopy methods often provide a limited view of the angle without visual documentation. The NIDEK GS-1 Gonioscope was the first device to automatically provide 360° color imaging of the anterior chamber angle with a 16x mirrored facet lens [10,11,12].

Gonioscopy is commonly used to assess the postoperative positioning of chamber angle-based MIGS devices. The purpose of this study was to evaluate automated 360° gonioscopy for the detection and documentation of postoperative positional variation after Hydrus^®^ Microstent implantation. To our knowledge, this is the largest study of its kind.

## 2. Materials and Methods

The present study was conducted in accordance with the ethical standards of the Ethics Committee of the Medical Association of Westphalia-Lippe and the University of Münster. It adhered to the tenets of the Declaration of Helsinki. Informed consent and ethical approval were waived by the Ethics Committee of the University of Münster, North Rhine-Westphalia, Germany, due to the retrospective nature of this study in accordance with Section 6 of the Health Data Protection Act of North Rhine-Westphalia (GDSG NRW). This study was conducted at the Department of Ophthalmology, University Hospital Münster, Münster, Germany.

**Figure 1:**
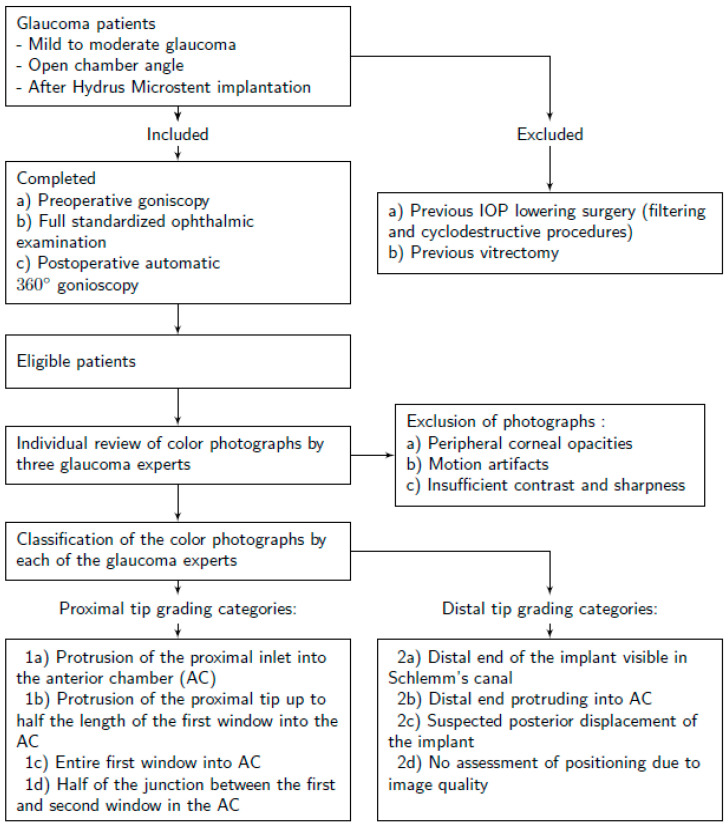
Study design.

The study design is illustrated in Figure 1.

For this study, we analyzed electronic medical records (FIDUS, Arztservice Wente GmbH, Darmstadt, Germany) of patients with primary open-angle glaucoma (POAG) and pseudoexfoliative glaucoma (PEX-G) who underwent Hydrus^®^ Microstent implantation combined with phacoemulsification or alone.

The inclusion criteria for Hydrus^®^ Microstent implantation was the presence of mild to moderate glaucoma with an open angle (open-angle glaucoma/pseudoexfoliation glaucoma). Patients with angle closure, traumatic, malignant, uveitic, and neovascular glaucoma, and those with chamber angle anomalies were excluded.

Patients had to have available data on postoperative IOP, information on postoperative antiglaucomatous medications, and any additional IOP-lowering procedures and ocular adverse events following Hydrus^®^ Microstent implantation. Patients who had previously undergone IOP-lowering procedures with filtering and cyclodestructive methods and those who had undergone vitrectomy were excluded.

All patients underwent a standardized ophthalmic examination including refractive and anterior segment examination, fundoscopy, Goldmann applanation tonometry, and perimetry. Perimetry was performed with the standard 30-2 Swedish Interactive Threshold Algorithm (SITA fast) program using the automated Humphrey Visual Field Analyzer II (HFA II, model 750, manufactured by Carl Zeiss Meditec AG, Jena, Germany).

Preoperative gonioscopy was performed to rule out angle abnormalities (synechiae, rubeosis, angle closure), which was necessary to ensure eligibility for Hydrus^®^ Microstent implantation. The procedures were performed by three glaucoma surgeons using either a combined cataract and MIGS procedure or a single MIGS implant in cases of previous cataract surgery and resulting pseudophakia. In the case of cataract surgery, phacoemulsification was performed through a clear corneal incision. A detailed description of the procedure can be found in the freely available manual. During implantation, a gonioprism (Alcon, Fort Worth, Texas, USA) was used to visualize the anterior chamber angle. Miosis facilitates implantation. Implantation is performed with a hand-held delivery system after the cannula is inserted through the clear corneal incision into the anterior chamber. The microstent can be advanced through the trabecular meshwork into Schlemm’s canal using a tracking wheel on the cannula. The implant passes through Schlemm’s canal during implantation. At the end of implantation, only the inlet of the implant should be visible in the anterior chamber. If properly positioned, the stent will appear opaque through the trabecular meshwork in the canal. A shiny stent is anterior to the trabecular meshwork. It is recommended that the inlet be positioned in the anterior chamber.

If the stent is not visible during implantation, it may be posterior to Schlemm’s canal. In this case, repositioning is recommended.

**Figure 2:**
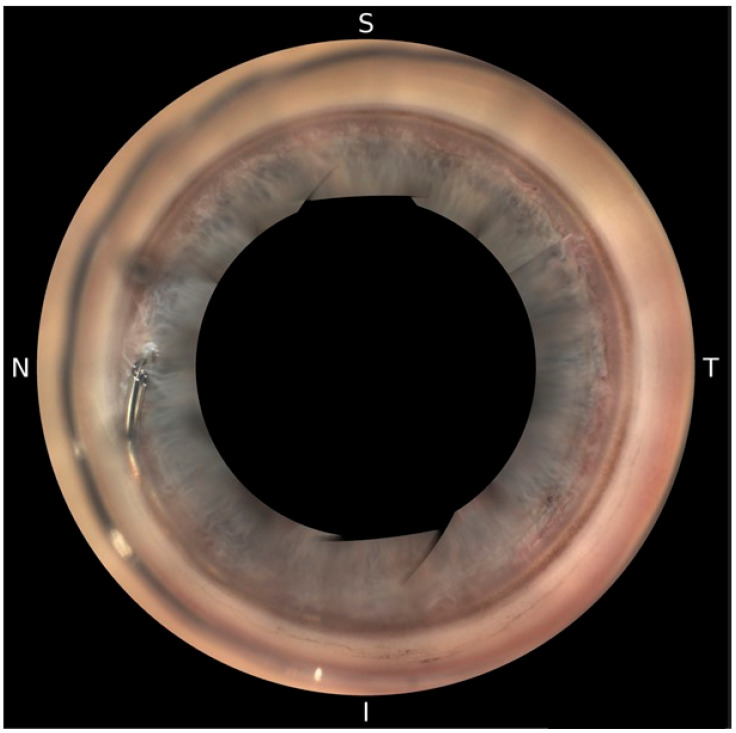
Hydrus^®^ Microstent visualized with NIDEK GS-1 Gonioscope.

**Figure 3:**
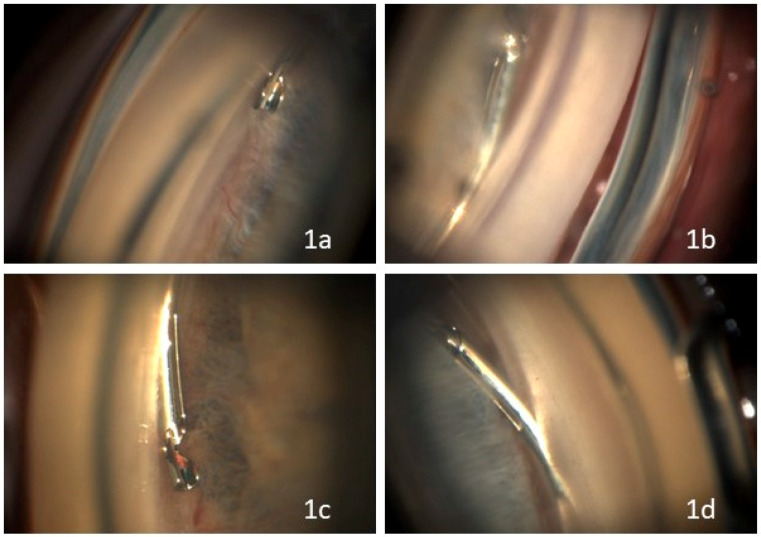

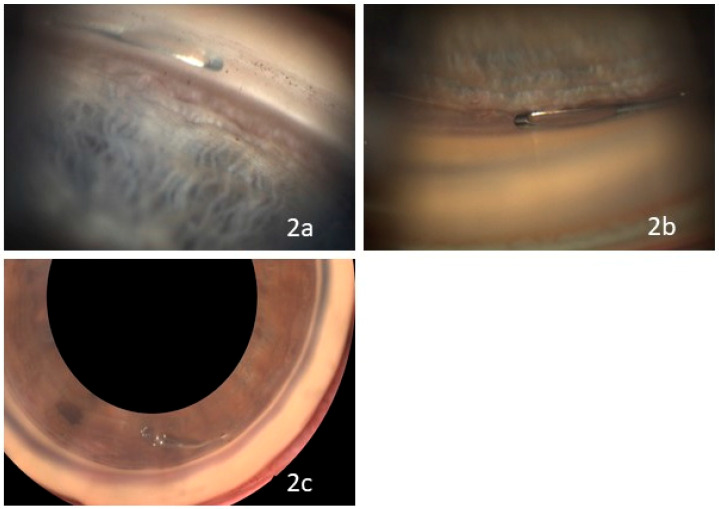
Visualization of the postoperative position of the proximal and distal tips of the Hydrus^®^ Microstent using automated 360° gonioscopy. Only the proximal tip is visible in the anterior chamber (**1a**). Half of the first window of the stent (viewed from the proximal end with the inlet in the anterior chamber) protrudes into the anterior chamber (**1b**). The entire first window is in the anterior chamber (**1c**). At least half of the bridge, called the spine, between the first and second windows is in the anterior chamber (**1d**). The distal rounded tip is clearly visible behind the trabecular meshwork within Schlemm’s canal (**2a**). The distal tip protrudes into the anterior chamber (**2b**). The distal stent is not visible through the trabecular meshwork, suggesting a posterior location (**2c**).

**Figure 4:**
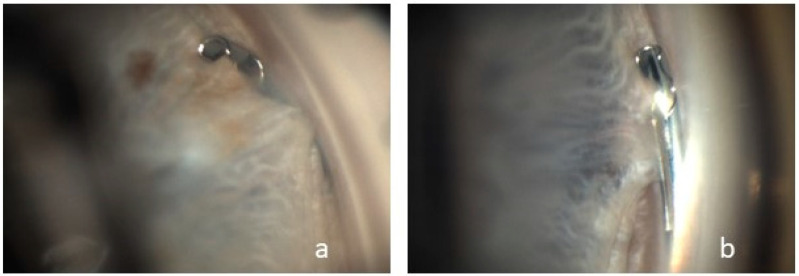
Visualization of two cases (**a**,**b**) of peripheral synechiae between the iris and the proximal tip of the Hydrus^®^ Microstent.

Postoperatively, patients underwent automated 360° gonioscopy to visualize the postoperative positioning of the Hydrus^®^ Microstent (Figure 2, Figure 3 and Figure 4). The examination was performed by two experienced glaucoma specialists. Immediately after the examination, they reviewed the individual images from the 16 image directions. In addition to the individual images, 360° images of the chamber angle were obtained. Implant position was independently assessed by three experienced glaucoma specialists using the single images and the 360° composites. The postoperative position of the implant was described according to the following staging: 1a describes a protrusion of the proximal inlet into the anterior chamber. The proximal inlet (Figure 3, 1a) of the implant continues uninterrupted into a first recess, referred to here as the “first window”. This was followed by three more apertures (windows 2–4). Stage 1b describes a protrusion of the proximal end of the implant up to half the length of the first window into the anterior chamber. In Stage 1c, the entire first window is in the anterior chamber, and in Stage 1d, half of the connection (“spine”) between the first and second windows is also in the anterior chamber.

The assessment of the distal tip was divided into categories 2a–d. In Stage 2a, the distal end of the implant is visible in Schlemm’s canal; in Stage 2b, it protruded into the anterior chamber. In Stage 2c, the entire chamber angle is clearly visible on gonioscopy, but the distal tip of the implant could not be visualized, suggesting the posterior displacement of the implant out of Schlemm’s canal. Stage 2d contains all cases in which the image quality does not allow assessment of the distal position. Images that could not be classified as class 1 or 2 due to anatomic conditions such as peripheral corneal opacities or motion artifacts were excluded from the analysis. Only images with sufficient contrast and sharpness to visualize the structures of Schlemm’s canal were included.

## 3. Statistical Analysis

Statistical analysis was performed using GraphPad Prism 10 (GraphPad Software, Boston, MA, USA) with data extracted from the electronic patient record (FIDUS, Arztservice Wente GmbH, Darmstadt, Germany). Normal distribution was tested using the Shapiro–Wilk test. The Wilcoxon matched-pairs signed rank test was used to analyze IOP and the number of drops used over time (nonparametric, dependent samples). Statistical significance was defined as a *p*-value less than 0.05.

## 4. Results

A total of 23 eyes were included in the study. The demographics of the study cohort are shown in Table 1. In six cases, the Hydrus^®^ Microstent was implanted as a stand-alone procedure. All eyes showed a reduction in IOP and a reduction in the number of antiglaucomatous eye drops compared to preoperative values (Table 2). In the subgroup analysis of Stage 2c patients, there was also a statistically significant reduction in IOP one month after surgery. There was no difference between eyes treated with a combination of cataract surgery and Hydrus^®^ Microstent implantation compared to stand-alone Hydrus^®^ Microstent implantation.

Analysis of the postoperative 360° gonoscopic images revealed the following findings: Interobserver variability in image interpretation and classification did not differ, as the structures defined for grading were clearly definable, except for the definition of distal dislocation. The final grading of the postoperative positions was in full agreement among all three subviewers. The distribution of the different implant positions is shown in Table 3. Figure 3 shows the different types of positions of the proximal and distal tip. In four implants only, the inlet was in the anterior chamber (1a). In 14 cases, approximately half of the first window of the stent (viewed from the proximal end with the inlet in the anterior chamber) protruded into the anterior chamber (1b). In three cases, the entire first window was in the anterior chamber (1c). In two cases, at least half of the bridge, called the spine, was visible between the first and second windows in the anterior chamber (1d). In no case was the second window (viewed from the proximal end with the entrance in the anterior chamber) visible in the anterior chamber.

In five cases, the distal rounded tip was clearly visible behind the trabecular meshwork within Schlemm’s canal (2a). In one case (2b), the distal tip was in the anterior chamber. In seven cases, the distal stent was not visible through the trabecular meshwork, suggesting a posterior location (2c). In 10 cases, the distal course could not be assessed (2d).

A subgroup analysis of groups 1a and 1b (n = 18) showed a statistically significant reduction in IOP at one and six months postoperatively compared to the baseline IOP. In this group, the number of antiglaucomatous eye drops was statistically significantly reduced at one month compared to the baseline. In the 1c and 1d groups (n = 5), there was no statistical reduction in IOP or the number of antiglaucomatous drops. Among implants with a presumed posteriorly displaced posterior end (2c, n = 7), two cases had only associated proximal inlets in the anterior chamber (1a). In five other cases, a grade 1b situation was found proximally. In all cases with grade 2c involvement (n = 7), there was a statistically significant reduction in IOP at one month postoperatively compared to the baseline.

Of the three PEX-glaucoma eyes, one eye belonged to group 1a and two to group 1b. Distally, the stent was visibly located in Schlemm’s canal in two eyes (2a); in one case, the distal position could not be assessed (2d). All three showed a reduction in IOP and a reduction in the number of antiglaucomatous eye drops over the observation period.

**Intraoperative and postoperative Adverse Events.**

In none of the cases in our cohort did any of the intraoperative events detailed in the instructions for use occur.

Postoperatively, hypertensive IOP occurred in four eyes. In all cases, IOP was well controlled with local and systemic IOP-lowering therapy. In none of the cases could a malposition of the stent explain the IOP elevation. In one patient, IOP elevation was suspected as a reaction to the administration of topical steroids. Only in one of the four cases could the IOP not be lowered to the target range and, therefore, a filtering glaucoma surgery was planned. Postoperative hypotension below 6 mmHg for one month was not observed in any of the cases.

Peripheral synechiae between the iris and the implant without obstruction was observed in four cases (Figure 3).

## 5. Discussion

The goal of minimally invasive glaucoma surgery (MIGS) devices is to lower intraocular pressure sufficiently. They are particularly suitable for patients with mild or moderate open-angle glaucoma and promise a lower risk profile compared to conventional methods such as trabeculectomy [4].

MIGS devices can be categorized by the mechanism by which they reduce IOP. In addition to the devices that drain aqueous humor suprachoroidally and those that filter under the conjunctiva and form a filtering bleb, there are also angle-based implants. Besides the Kahook Dual Blade, the ISTENT^®^ family of implants are prominent representatives of angle-based MIGS implants [13]. ISTENT inject^®^ and ISTENT inject^®^ W (Glaukos Corporation, Laguna Hills, CA, USA) are second and third generation angle-based bypass systems. The implants improve aqueous humor outflow through the trabecular meshwork into Schlemm’s canal. Postoperative IOP reduction, the reduction of antiglaucoma medications, and the safety of the product have been demonstrated in studies [14,15,16,17,18]. In their analysis of the postoperative positioning of ISTENT inject^®^ microstents, Gillmann et al. presented anterior segment optical coherence tomography (AS-OCT) as an evaluation method in addition to gonioscopy. In their postoperative analysis, more implants were visible with AS-OCT than with gonioscopy. They also examined the postoperative diameter of Schlemm’s canal and found that it was enlarged in patients after ISTENT^®^ implantation compared to the control group (308.7 ± 197.4 µm versus 126.9 ± 60.3 µm) [15,19]. In a previous study, we evaluated the postoperative visualization of ISTENT inject^®^ using automated gonioscopy. Only 14.3% of the implants could not be visualized [20]. In addition to automated gonioscopy, we believe that AS-OCT is a very interesting method to analyze the postoperative positioning of the Hydrus^®^ Microstent, especially for devices that cannot be visualized by gonioscopy due to a presumed posterior dislocation. Due to its long course in Schlemm’s canal, we assume good visualization and dilation of the canal.

The Hydrus^®^ Microstent is a MIGS implant approved for the treatment of mild to moderate primary open-angle glaucoma in adults in combination with cataract surgery to lower intraocular pressure.

The randomized, controlled, multicenter HORIZON study compared cataract surgery with cataract surgery combined with Hydrus^®^ Microstent implantation over five years [5,8].

A total of 187 eyes underwent cataract surgery and 369 eyes underwent combined surgery. At five years, there were significantly more eyes in the combination group with IOP reduction to 18 mmHg without medication or less than in the control group. The combination group was also more likely to achieve an IOP reduction of 20% or more without medication [8]. In our cohort, a reduction in IOP and a reduction in the use of IOP-lowering medications were also achieved over the observation period.

Gonioscopy is used for the visualization and examination of the iridocorneal angle. It is part of a complete eye examination, especially in glaucoma patients, and has both diagnostic and therapeutic implications [9]. The performance and the results of the gonioscopy are dependent on the examiner. It takes a lot of practice and training to master manual gonioscopy [21]. Only a portion of the corneal angle can be examined at a time.

Because different examiners may perceive and evaluate angle structures differently, the interpretation of the findings obtained during the examination is subjective. A joint assessment of the chamber angle is almost impossible because the gonioscopy is performed by only one person at a time. Important treatment decisions depend on this assessment. The fact that the chamber angle is photodocumented by automated gonioscopy means that a joint assessment can be performed by multiple health care professionals. The time of the assessment is independent of the time of the examination. Another advantage is that structures in the anterior chamber that are located in different planes can be visualized by different focusing [10,11,12].

However, in our experience, even with automated gonioscopy, training is necessary to obtain reliable results. In addition, the use of automated gonioscopy is still dependent on patient cooperation. As with the use of handheld devices, a clear view of the anterior chamber is necessary to obtain meaningful images. Motion artifacts also play a role, resulting in the shifting of the individual images that make up a 360° image. Corneal opacity, whether postoperative due to edema or other changes, complicates the assessment of the anterior chamber angle, as evidenced by the number of our patients in whom the anterior chamber angle implant could not be fully assessed.

A number of intra- and postoperative complications have been described. The Horizon study defined adverse events related to stent implantation. These included postprocedural malapposition, peripheral anterior synechiae with partial or complete device occlusion, and device removal. The cumulative number of events for the occurrence of postprocedural malapposition was 1.4 at two-, three-, four-, and five-years postprocedure.

Peripheral anterior synechiae with device occlusion was reported as a cumulative event in 3.5 cases at two years and 5.4 cases at five years. As in the prospective, randomized study by Pfeiffer et al. and in the COMPARE study by Ahmed et al., we were able to demonstrate the formation of focal peripheral anterior synechiae, which is also listed in the Hydrus^®^ Manual as the most frequent complication of combined MIGS and cataract surgery with a prevalence of 40% [5,22,23]. Pfeiffer et al. followed their patients for 2 years and reported synechiae in 12% of their patients in the first year, increasing to 18.8% in the second year [22]. In our study with a shorter follow-up, there were four cases in which we observed peripheral synechiae in the proximal inlet area. The patients in our study cohort in whom synechiae formation was visualized were examined by 360-degree gonioscopy at a mean interval of 278.25 days, suggesting that synechiae formation may increase in the postoperative course. In addition to synechiae formation, Ahmed et al. described tissue adhesions to the iris as a postoperative side effect, which in one patient had to be removed by yttrium–aluminum–garnet laser treatment. In none of these cases did the IOP evolution indicate implant occlusion [8]. None of the eyes included in our study required secondary surgical intervention.

In the IVANTIS instructions for use, malposition was defined as an adverse event if the position required a follow-up procedure for repositioning or explantation. This included corneal endothelial contact, central endothelial cell loss >30%, device obstruction, or chronic inflammation or irritation [5,8]. Based on this definition of malposition as an adverse event, no such event was identified in our cohort. The Hydrus^®^ Microstent instructions for use lists intraoperative adverse events. In the HORIZON study, an adverse event occurred in 3.8% of the cases. The most common of these was a hyphaema resulting in decreased vision during the procedure (1.1%). Device malposition, Descemet’s membrane detachment, iris prolapse, and wound incarceration were also reported. In our cohort, there were no intraoperative adverse events in significantly fewer cases.

In addition, the proximal end of the Hydrus^®^ Microstent is less parallel to the chamber angle in all cases in our study compared to the 24-month images in the Horizon study but extends slightly further into the anterior chamber. In only one case did the most distal end protrude into the anterior chamber (Figure 3; Table 3).

The microstent is made of nitinol, an alloy of nickel and titanium. According to the manufacturer, the material offers flexibility, strength, and biocompatibility. In addition, the material is elastic enough to be inserted into and support Schlemm’s canal. During the manufacturing process, the microstent is heat-set to match the curvature of Schlemm’s canal. During implantation, the proximal inlet is placed in the anterior chamber to ensure aqueous humor drainage through the windows of the implant.

Laroche et al. suggest a postoperative change in stent configuration as a possible explanation. They describe that the curvature of the stent corresponds to a circle with a diameter of 12 mm, while the mean diameter of the cornea is 11.74 mm. The nitinol used is a flexible material that, according to the manufacturer, has both shape memory and flexibility [5].

As described by Laroche et al. in their case series, it can be assumed that a greater curvature of the Hydrus^®^ Microstent compared to the course of Schlemm’s canal into which it regresses causes the distal end to protrude into the anterior chamber in the cases described in our study [24]. Assuming that the distal end was still in the canal intraoperatively, an increase in curvature in the postoperative course would be expected. This may also explain a slight incision of the chamber angle as a subtle goniotomy through the proximal end, especially in cases where the proximal tip protrudes into the anterior chamber (grade 1c + d) (Figure 3).

The patients in our study had an axial length between 22.35 mm and 25.42 mm. We did not find a correlation between increased axial length and postoperative change in stent position.

Across all groups, there was a statistically significant reduction in postoperative IOP as well as a reduction in the number of daily antiglaucomatous eye drops. The question of the relationship between IOP reduction and drop reduction and the postoperative position of the implant in the chamber angle is very interesting in addition to the general assessment of the position by automated gonioscopy. A larger number of cases in all defined classes is necessary to provide a sufficient answer and is currently being planned. Patients with POAG were also included in the pivotal studies. The three patients with PEX- glaucoma included in this study also showed a clinical reduction in IOP and a reduction in the number of postoperative eye drops. In the future, it would be desirable to study more patients with PEX-glaucoma to obtain statistically verifiable evaluations of IOL positioning and postoperative outcomes. This is particularly interesting because the included eyes with PEX-glaucoma showed IOP reduction in the study but were not included in the pivotal trials.

Contraindications to implantation include changes in the iridocorneal angle. These include angle closure glaucoma and post-traumatic conditions, the presence of neovascularization, and congenital changes in the angle.

None of these baseline findings were present in the patient cohort. However, anatomical changes in Schlemm’s canal that cannot be assessed preoperatively may lead to changes in implant position. Although no intraoperative resistance was observed during implantation, subtle adhesions and herniations in Schlemm’s canal cannot be excluded.

Although this is the largest cohort studied after Hydrus^®^ implantation using automated gonioscopy, a limitation of the study is the relatively small number of eyes included. After completion of this feasibility study, a larger number of eyes would be useful to create subgroups. These could be grouped according to the location and type of glaucoma.

Another limitation is the retrospective design of the study, as gonioscopy was performed at different times after surgery. In order to standardize variables in the future, prospective studies are desirable. As this is both a relatively new surgical technique and a new imaging modality, future follow-up studies with longer observation periods are desirable and planned.

## 6. Conclusions

The study results show a reduction in IOP after Hydrus^®^ Microstent implantation. Automated 360° gonioscopy proved to be a useful tool to verify and document the postoperative position of the implant.

We have demonstrated variations in the postoperative position of the Hydrus^®^ Microstent. Attempts to explain this have focused on anatomic conditions, stent configuration, and the interaction between the two. This study demonstrated that the Hydrus^®^ Microstent can effectively lower IOP even in the presence of post-op positional alterations. Positional changes did not require device explantation in any of the cases evaluated.

Follow-up studies with a larger number of patients and a longer follow-up period are currently being planned to track changes in position beyond the 12th month after implantation.

## Figures and Tables

**Table 1:** Study cohort demographics and baseline characteristics. Continuous variable data are reported as mean (±standard deviation) or median (25th percentile; 75th percentile), depending on data distribution.

Patients/Eyes (n)	17/23
Diagnosis	
POA-Glaucoma	20/23 (87.0%)
PEX-Glaucoma	3/23 (13.0%)
Age (years)	69.5 ± 7.8
Sex (m/f)	3 (17.6%)/14 (82.4%)
Visual field MD (db)	−5.0 [−10.6; −2.8]
Baseline IOP (mmHg)	20.0 [16.0; 24.0]
Number of antiglaucoma eye drops at baseline	2 [1; 3]

POA-glaucoma, primary open-angle glaucoma; PEX-glaucoma, Pseudoexfoliative glaucoma; m, male; f, female; IOP, intraocular pressure; MD, mean deviation.

**Table 2:** IOP and antiglaucomatous eye drops in the observation period. Data on continuous variables are reported as the median (25th percentile; 75th percentile) based on the data distribution.

IOP in mmHg	Baseline vs.	20.0 [16.0; 24.0]	*p*-value
	1 month	14.0 [11.0; 15.0]	<0.0001
	6 months	14.0 [11.5; 16.5]	0.0234
	12 months	14.0 [12.25; 18.5]	0.1172
Daily antiglaucomatous eye drops (n)	Baseline vs.	2 [1; 3]	
	1 month	1 [1; 2]	0.0156
	6 months	1 [1; 2]	0.1562
	12 months	2 [1.25; 2]	0.6562

IOP, intraocular pressure.

**Table 3:** Assessment of the position of the Hydrus^®^ Microstent in the chamber angle.

Position Assessment	Eyes (n)
Position of the proximal tip	
1a	4/23 (17.4%)
1b	14/23 (60.9%)
1c	3/23 (13.0%)
1d	2/23 (8.7%)
Position of the distal tip	
2a	5/23 (21.7%)
2b	1/23 (4.3%)
2c	7/23 (30.4%)
2d	10/23 (43.5%)

n, number.

## Data Availability

The data that support the findings of this study are available on request.
